# UAS imagery reveals new survey opportunities for counting hippos

**DOI:** 10.1371/journal.pone.0206413

**Published:** 2018-11-14

**Authors:** Julie Linchant, Simon Lhoest, Samuel Quevauvillers, Philippe Lejeune, Cédric Vermeulen, Jean Semeki Ngabinzeke, Basile Luse Belanganayi, Willy Delvingt, Philippe Bouché

**Affiliations:** 1 TERRA Research Center, Central African Forests, Gembloux Agro-Bio Tech, University of Liège, Gembloux, Belgium; 2 Forest Ressources Management, Department BIOSE, Gembloux Agro-Bio Tech, University of Liège, Gembloux, Belgium; 3 Département de Gestion des Ressources Naturelles, Faculté des Sciences Agronomiques, Université de Kinshasa, Kinshasa, République Démocratique de Congo; 4 Faculté de Gestion des Ressources Naturelles Renouvelables, Département des Eaux et Forêts, Université de Kisangani, Kisangani, République Démocratique du Congo; 5 Professeur émérite, Gembloux Agro-Bio Tech, University of Liège, Gembloux, Belgium; Institute of Animal Science, CZECH REPUBLIC

## Abstract

**Introduction:**

The common hippopotamus *Hippopotamus amphibius* L. is a vulnerable species that requires efficient methods to monitor its populations for conservation purposes. Rapid evolution of civil drones provides new opportunities but survey protocols still need development. This study aims to determine the optimal flight parameters for accurate population estimates. A second objective is to evaluate the effects of three environmental factors: wind speed, sun reflection and cloud cover.

**Method:**

We estimated the population of two main hippo schools (Dungu and Wilibadi II) located in Garamba National Park in Democratic republic of Congo. Eight observers reviewed 252 photos taken over the Dungu school, representing a total of 2016 experimental units. A detection rate and a level of certainty were associated with each experimental unit, and five parameters were related to each count: flight height, three environmental parameters (sun reflection on water surface, cloud cover, and wind speed), and observers’ experience.

**Results:**

Flight height reduced the observers’ confidence in their detection ability, rather than the detection itself. For accurate counts of large groups an average height of 150 m was shown to be a good compromise between animal detection without zooming in and the area covered in one frame. Wind speed had little influence on the counts, but it affected the performance of the UAS. Sun reflection reduced the detection rate of hippos and increased level of certainty, while cloud cover reduced detection rates slightly. Therefore, we recommend flying when the sun is still low on the horizon and when there is little cloud, or when cloud cover is light and even. This last point reinforces our recommendation for flights early in the day. The counts also showed large differences between groups of inexperienced and experienced observers. Experienced observers achieved better detection rates and were generally more confident in their detection. Experienced observers detected 86.5% of the hippos on average (confidence interval = ±0.76%). When applied to data from the second site, the detection was 84.3% (confidence interval = ±1.84%). Two correction factors were then calculated, as the inverse of the detection rate, based on the estimated number of hippos present during one flight (Factor 1) or in the general population respectively (Factor 2). Factor 2 especially was consistent with previous studies using traditional aerial counts (1.22 *vs* 1.25). Factor 2 was found to be appropriate for use by experienced observers. These results confirm the use of correction factor 2 for hippo surveys, regardless of the study site, as it accounts for hippo behavior. Optimum counting and cost efficiency were achieved with two trained observers counting 7 pictures.

**Conclusion:**

This study is a promising approach for routine surveys of the hippopotamus which is a species usually ignored in wildlife counts. Drone technology is expected to improve rapidly; therefore UAS could become a very useful and affordable survey tool for other species requiring specific monitoring.

## Introduction

The common hippopotamus *Hippopotamus amphibius* L. is found throughout sub-Saharan Africa [1] where it is valued not only for tourism (game viewing and sport hunting), or cropping (bush meat), but also for its ecosystem services such as fertilizing its aquatic habitat [2,3]. IUCN classifies the species as "vulnerable" (IUCN 2016, iucnredlist.org). The continental population is estimated to have declined by 7 to 20% between 1998 and 2008 [4]. The main threats to hippopotamuses are poaching and habitat loss [5,6]. In DRC (Democratic Republic of Congo), populations fell to 5% of their historic levels with the last significant remaining populations in Virunga and Garamba National Parks [4,6].

Counting hippos during the day in an aquatic environment is difficult [7], and they are often ignored during wildlife surveys [2,3,8]. The uneven distribution of hippopotamuses in a river can be partially explained by segments with sufficient depth to completely submerge the adults, and by the distribution of nearby adequate pasture [9,10]. Hippos gather in groups of between 10 to 200 individuals [1,7,11–14]. The frequency distribution of group sizes is far from normal and very asymmetric. The average group size varies positively with the hippo density in the sector [7]. The largest groups were found in big ponds and densely populated lakes [7,11–14].

Those groups named "schools" in the literature are described as having a dominant male surrounded by females and young individuals. Other males stay together separated from the main group [1,15,16]. The intensity of activity in a school varies during the day. Large ponds and lakes serve as refuges against predators [lions, hyenas, leopards and humans][7].

One needs an easy, low-cost, accurate, precise and reproducible method when estimating hippo populations [7]. Hippos have been counted on foot, from boats, and from the air using direct observations or aerial pictures [7–9,16–19]. Different methods can also be used in combination to gain precision [7,20]. The dry season is the best time because hippos retreat to a limited number of remaining shallow pools with reduced turbidity. It is then possible to spot submerged hippos from the air [8].

From ground and boat, observers follow lake shores, drawdown zones of reservoirs and riverbanks while counting all hippopotamuses they see [7,19]. Observer efficiency is an important source of bias leading to underestimates especially where hippos live in large groups [7]. Lack of precision in such counts is correlated with the group’s size and with visibility constraints: while counting a hippo group, the almost flat vision angle hides the view of animals in the back, and some parts of the river may be completely out of sight or inaccessible [7]. Therefore, these methods produce highly variable results. Boat counts are very slow and do not cover long rivers well [9].

Aerial counts can be performed from light aircraft, ultralight or helicopter [7,8,18,21,22]. Ultralights and helicopters can fly at lower speed and are more manœuverable [18]. Aircraft follow the streams at low speed (90 to 120 km.h^-1^) and height (50 to 90 m). However, hippos react to the passage of aircraft by raising their heads and then diving [7].

Previous methods have been shown to give under-estimates for large populations [7]. Estimates can be improved by taking aerial photographs of populations of a few hundreds to thousands of hippos. Pictures are taken over the whole surveyed area and then sampled randomly. Hippopotamuses in the pictures are counted, and assuming the sample is large enough, a total count can be extrapolated to the whole study area [7]. However, optimal weather conditions (time of the day, cloud cover and wind speed), pictures of high quality (sharpness and resolution) and trained observers are required to produce reliable estimates [7].

Delvingt [7,23] developed a density estimation method taking into account several parameters: group stability, daily diving rhythm, the influence of waves (especially on the lakes), and the effect of disturbing the hippos. It showed that group stability was assured for a period of 2 to 5 days. The diurnal rhythm showed that hippos dive more often during the middle of the day [9].

Aerial surveys cover large areas in a short time and remain the best alternative for counting large mammals in savannahs [18]. However, they are very expensive and depend on the availability of qualified teams and pilots, appropriate fuel and reliable well-maintained aircraft [24–28]. Human and material resources are often limited especially for most wildlife administrations in developing countries [29,30]. Wildlife monitoring often depends on external funding that is not always available, causing gaps of 10 to 25 years between successive counts [30,31]. Consequently a species can collapse during the interval without appropriate management action being taken. Finally, aerial surveys are dangerous operations, particularly when flying over remote areas at low altitude [32].

Because of the limitations of traditional aerial methods, the recent development of unmanned aerial systems (UAS; more commonly referred to as "drones") for civil applications triggered new initiatives to find alternatives. Indeed, the new technology presents some advantages that could make it a valuable new tool for wildlife surveys [27,30,33].

The main advantage of UAS is their ability to fly slowly at low altitudes while the operators remain safe. They can survey areas too difficult for ground surveys, such as wetlands, or challenging topography without human risk [34–36]. UAS, especially fixed-wings, are usually much quieter compared to manned aircraft, and may present less disturbance risk depending on the species being studied. Therefore UAS could reduce the risk of biased counts because animals are less likely to flee or hide. UAS capture imagery, along with the recorded flight parameters, giving geo-referenced data that can be used at diverse spatiotemporal scales [24,35].

Finally, while professionally manufactured UAS are still expensive, running costs are lower than for aircraft [25,27,37].

UAS promise new opportunities but standardized survey protocols must still be developed. This study aims to determine the optimal parameters for estimating accurate hippo population sizes with UAS. Specific objectives include (i) the evaluation of flight parameters optimizing the detection and visibility of hippos, such as flight height and weather conditions (sun reflection on water surface, cloud cover, and wind speed), (ii) the assessment of observer effect; and (iii) the estimation of correction factors applicable to animal numbers counted on one or more pictures to improve the final estimate of the population size.

## Material and methods

Field work was permitted by the Institut Congolais pour la Conservation de la Nature (ICCN) and the Government of the Orientale Province.

### Study area

**Fig 1:**
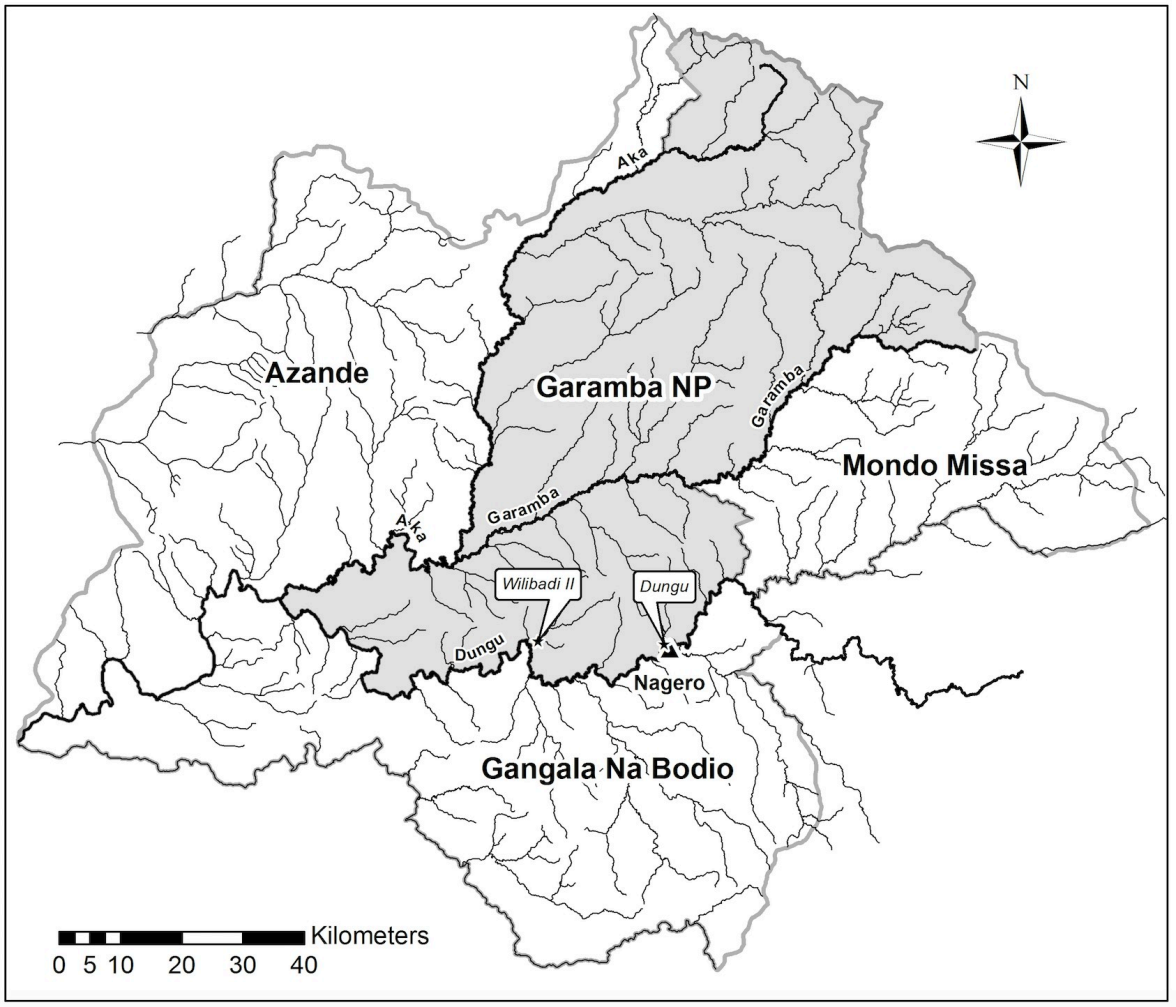
Map of Garamba National Park (DRC). The two study sites, Dungu and Wilibadi II hippo schools, are located in the southern area.

Garamba National Park (hereinafter GNP) is located in North-Eastern Democratic Republic of Congo (hereinafter DRC), at the border with South Sudan (3°45'-4°41'N. 28°48'-30°00'E) and covers more than 5100 km^2^ (Fig 1). A mosaic of grass and tree savannahs covers the park, and numerous small rivers with grassland valleys and papyrus swamps dissect it. The climate is tropical semi-humid type with the rainy season going from March to November (1500 mm of rainfall). There is a short dry season between December and February [38,39].

GNP's large open savannah is home to some of the last populations of endangered species in DRC such as elephants (a unique population representing a hybrid form between the forest and savanna species, *Loxodonta cyclotis* and *L*. *africana*) [40], giraffes (*Giraffa camelopardalis congoensis*), buffaloes (*Syncerus caffer*), hippopotamuses (*H*. *amphibius*), lions (*Panthera leo*), and various antelopes [38,39].

From the 1970s the park suffered increasing poaching pressure that was aggravated by armed conflicts in the region. Animal populations dropped to critical levels. However, a large population of hippos still survived in the park. The last published count, using different methods, reported over 2500 individuals [41].

We collected two sets of images in May (the rainy season) over two of the main hippo schools: Wilibadi 2 and Dungu, named after their respective river locations (Fig 1). The two sites lie in southern GNP, which has suffered less poaching.

### Unmanned aerial system

We used the Falcon UAS to collect the images (now discontinued but information sheet available at https://www.doi.gov/sites/doi.gov/files/uploads/Falcon Fixed-Wing Data Sheet.pdf). This small electrically powered fixed-wing UAS has a wingspan of 2.5 m and weighs 6 kg. It has an endurance of around 1 hour with an average speed of 50 km/h. The small UAV was equipped with an APM autopilot (http://firmware.eu.ardupilot.org). Flight plans and control of the UAS were managed from the ground control station with the open source software Mission Planner (http://firmware.eu.ardupilot.org).

The drone was equipped with a Sony Nex7 digital still camera (6000x4000 pixels) mounted with a 16mm pancake lens, producing RGB true color images, and two small video cameras for live transmission during flight. The payload was not mounted on a gimbal and therefore photo orientation is related to the plane’s orientation.

### Data acquisition

Before the survey we assessed the potential for disturbance of the Falcon. The Falcon is very silent and can only be noticed and heard when low and directly above the head. We flew passes over the school down to a 20 m flight height without recording strong reaction from the hippopotamus. No diving was recorded following the passes and few animals looked up. The shape and shadow of the UAS is similar to a large bird, which could explain the absence of reaction.

We conducted a total of 10 flights. Eight were above Dungu school between the 1^st^ and 10^th^ of May 2015 to evaluate the effect of different parameters. The last two flights were on 29^th^ of May above Wilibadi II school to compare data and to validate the method.

Most flights were in the early morning (6:30–8:30, and sometimes up to 12:30) when most hippos gather at the surface [7]. For each flight, weather conditions, takeoff and landing times were recorded.

**Fig 2:**
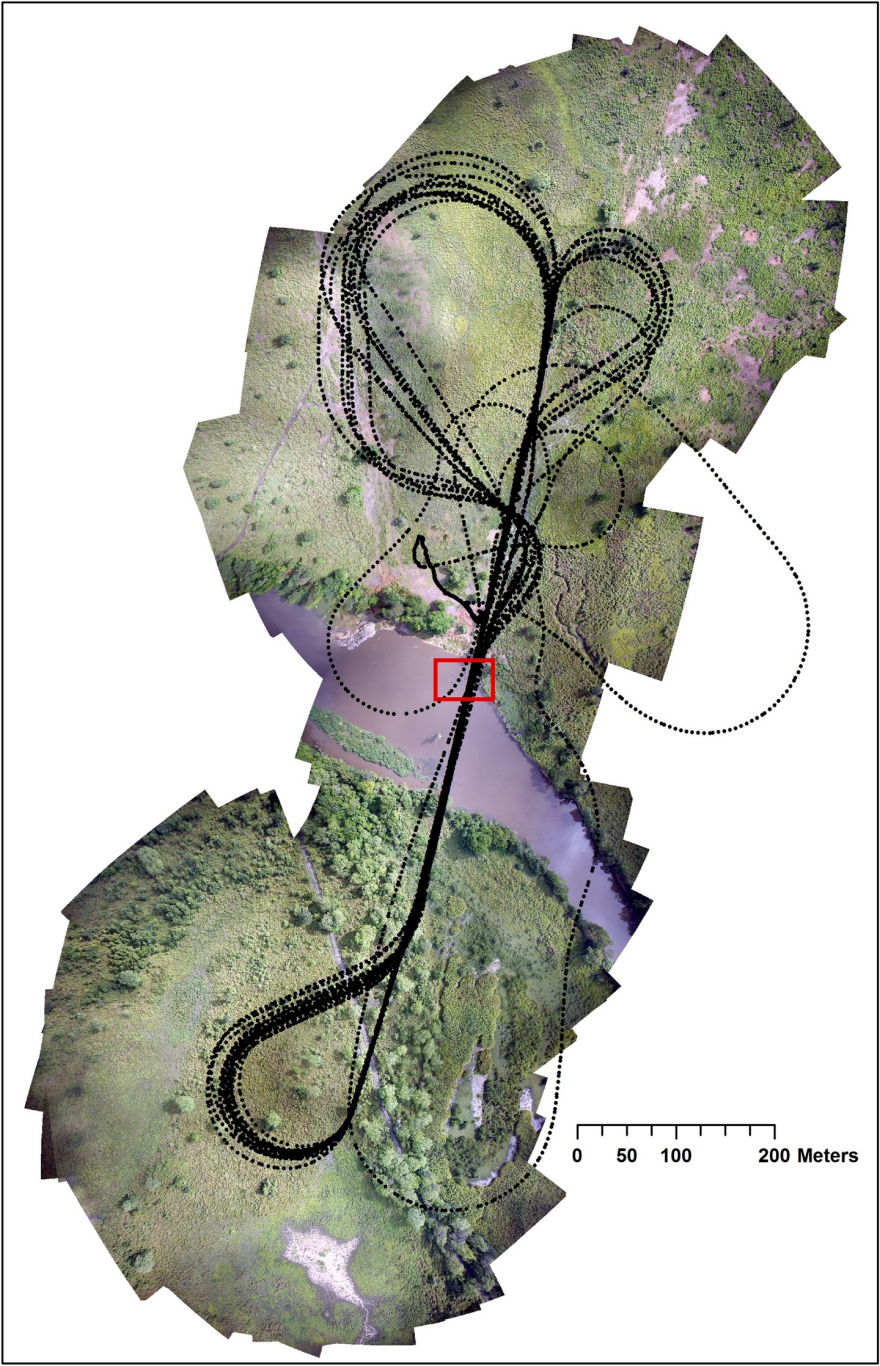
Flight plan tracking over the hippo school of Dungu. Black dots represent GPS points taken constantly during the flight and the red rectangle shows the location of the hippo school.

The UAS passed several times above the hippo schools (Fig 2). The initial flying height was 40 m. It was increased by 20 m every 2 or 3 round-trips (4 to 6 passes) up to a maximum of 140 m. The minimum of 40 m is a safe height, allowing a short reaction time in case of unplanned landing. It was also the minimum altitude needed to cover the entire school (at 40 m a photo covers a strip of 47.6 m wide; 166.6 m at 140 m). Previous tests showed that 140 m is the upper limit for easily detecting animals on the imagery.

Because large groups are more easily detected, and high-resolution images allow magnification with a reasonable resolution, it appeared that flying at higher altitude than 40 m could be more efficient. Therefore, we chose different flight heights for the dataset validation. Two flights above Wilibadi II were at 4 passes at 50, 100, 150, 200, and 250 meters above the ground level (AGL).

The drone was set to take pictures every 20 m (1.5 photos/sec, maximum rate for the camera) for the flight duration.

### Data processing

#### Image selection and pre-processing

The flights over Dungu and Wilibadi II produced large sets of data: 15,905 and 2,769 pictures respectively.

To determine the location and altitude of the pictures, each flight had been geo-referenced using Mission Planner to match GPS position and orientation data of the UAV [42].

From the large number of images taken at Dungu school, we selected the clearest picture (maximal sharpness and minimal sun reflection) of each pass, making sure that the group was completely visible in the frame. Since the number of repetitions varied amongst flights, a constant number of 42 pictures was randomly taken for each flight height from the pool of previously selected photos. A total of 252 photos of the same hippo school in different experimental conditions was selected (Table 1).

**Table 1:** Distribution of the selected images.

	Class	Number of pictures
**Flight heights (m)**	40	42
	60	42
	80	42
	100	42
	120	42
	140	42
**Sun reflection (0, 1 or 2)**	0 (absence of sun reflection)	187
	1 (minor sun reflection)	54
	2 (significant reflection creating white spots masking part of the photo)	11
**Cloud cover (0, 1 or 2)**	0 (clear sky)	18
	1 (light cover/scattered clouds)	169
	2 (numerous large clouds or black sky)	65
**Wind speed (m.s**^**-1**^**)**	1	32
	2	101
	3	36
	4	18
	5	65

Number of images for each category of the different flight parameters.

Four flight parameters were then chosen for further analysis: flight height, sun reflection at water surface, cloud cover, and wind speed. For every image all these parameters were scored based on the quality of the parameter in Table 1. Ground operators assessed sun reflection and pictures were sorted under three discrete modes. Cloud cover was recorded during each flight and related to corresponding pictures. Finally, wind speed was measured for each flight. The average wind speed was recorded by the UAS when flying in straight lines. Wind speed ranged from 1 to 5 m.s^-1^.

### Hippo counts

**Fig 3:**
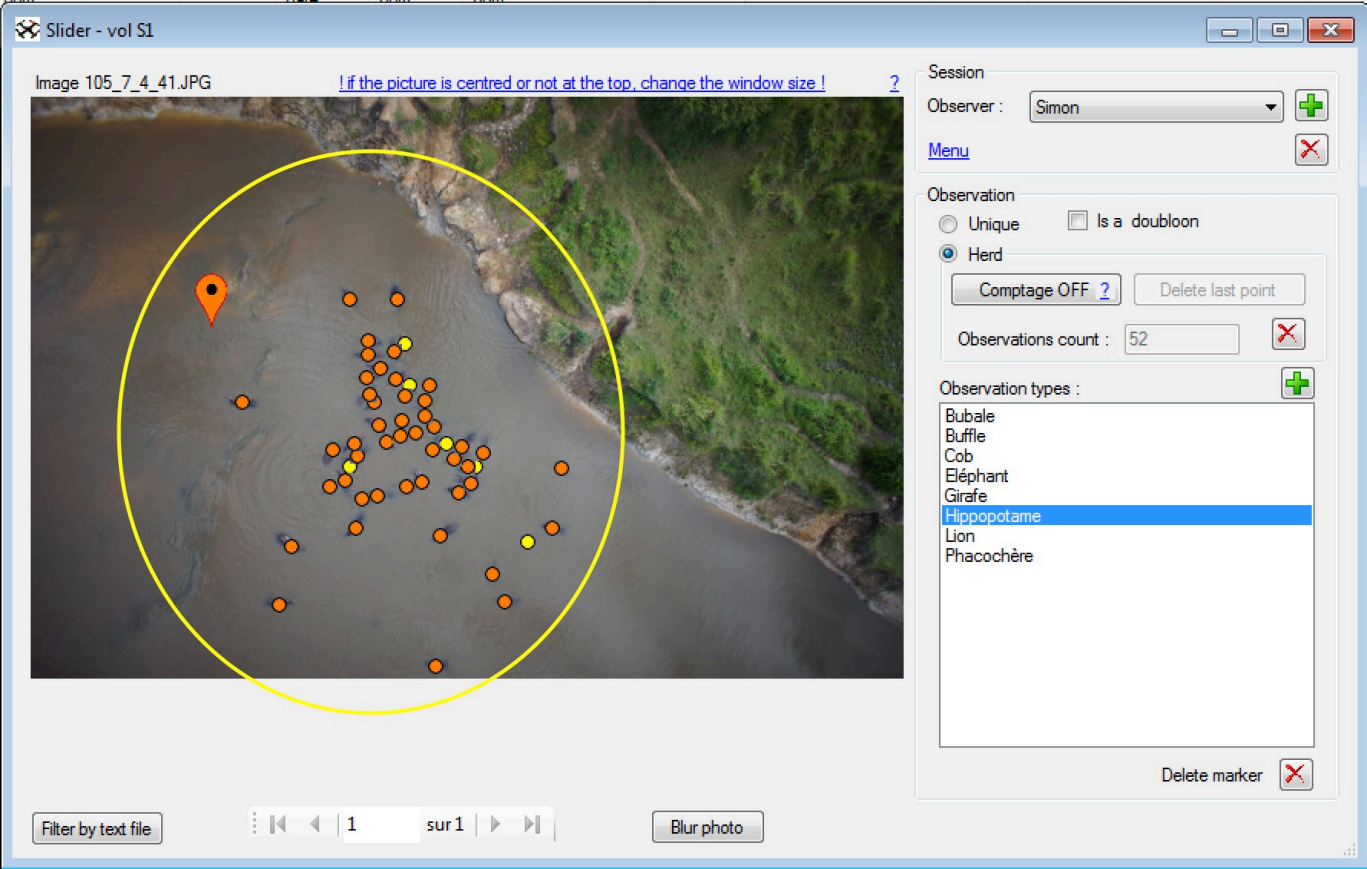
Interface of WiMUAS used to review the photos and detect animals. Orange points mark definite observations and yellow points possible identification. The yellow circle represents the area within which observers had to make their observations. The photo in this example was taken at the height of 41 m at 08:01. The environmental parameters recorded for this picture are cloud cover 1, sun reflection 0 and a wind speed of 3 m.s^-1^.

Observer effect was suspected to affect image-based population estimates [30,43]. Therefore, 8 independent observers counted the set of 252 images with the software WIMUAS (http://www.gembloux.ulg.ac.be/gf/outilslogiciels/VolDrone2016.7z) [42]. Three of them were the operators who conducted the flights and who were *a priori* familiar with animal detection from aerial pictures. The five others had no knowledge of the context and had never performed any animal counts before. After a briefing, observers counted hippos present in a limited area defined arbitrarily by a yellow circle (Fig 3) in order to avoid bias if hippos out of the main group were to be detected on larger photos. Observers were given the option of marking their observations as "definite" or "possible" by selecting the option "herd" in the software (Fig 3). The dataset was presented as a random succession of images from all the flights, and not in the order of successive passages, to avoid auto-correlation in recognizing the patterns of the hippos’ positions. Results from all observers were included in the same database for comparison.

### Estimation of the total number of hippos in the school

The total number of hippos present during one flight must be known in order to compare observer counts and calculate detection rates. Instead of taking the highest number observed for reference, we estimated the total number for each flight by using a succession of pictures and marking the movements of hippos.

**Fig 4:**
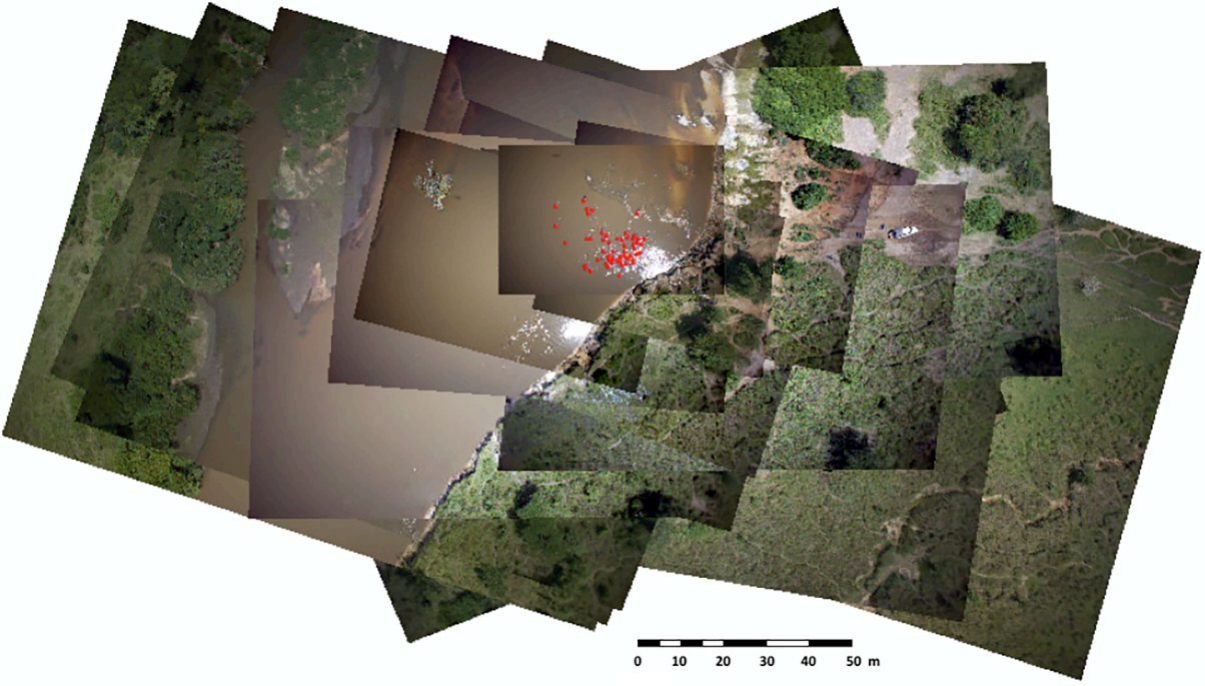
Overlap of 15 geo-referenced images. Red dots are the detected hippopotamuses recorded in the vector layer.

We selected one picture per UAV pass until we had 15 successive photos for each flight. The time between the first and the fifteenth photo was approximately 25 min guaranteeing that every hippopotamus emerged at least once because hippos dive for 3 to 5 min [9,16]. The 15 photos were geo-referenced and overlapped (Fig 4). The first photo being the largest we use it as the reference photo. The position was given by the onboard GPS and because the orientation was always almost nadir we did not rectify it further. We then overlapped the 14 other pictures using tie points (minimum 10 per image) with the georeferencing tool in Arcmap (ESRI) until all of them matched (with an average accuracy of 30 cm). We edited a vector layer in Arcmap and added a point for every hippo from the first picture. We then tracked animal movements on the next picture and moved points to match their new positions. Points were also added if a new hippo emerged, but were not deleted when they dived. The point layers were modified until we reached the estimated total number on the fifteenth image. This estimate was considered more accurate because it accounts for the movements of every hippo in the school.

### Optimization of flight parameters and influence of observer experience

We had a total of 2016 experimental units corresponding to the 252 photos reviewed by the eight observers (definite + possible observations). For each unit, a detection rate (DR) and a level of certainty (LC) were calculated. DR was obtained by dividing the total number of hippos by the estimated population for the specific flight. LC was calculated as the ratio between the number of definite observations to the total number counted by the observer. These rates are equivalent of what Chabot [26] defined as "detectability" and "visibility" and can be expressed as follow:

DR=tot.num/est.pop (1)

LC=def.obs/tot.num (2)

We fitted two mixed binary logistic regression models in order to describe the influence of the flight and environmental parameters on detection and certainty rates. Flight height and sun reflection varied among photos, while cloud cover and wind speed varied among flights. Since observer effect is an unavoidable parameter, and observers differed in experience, it is important to mitigate its influence on the population estimate. Observer effect was a dummy variable to distinguish experienced observers (0) from novices (1). The random effects were the observer, the photo and the flight. Including these random effects in the model accounted for the particular structure of our data set with correlations of the observations among observers, photo and flights. All explanatory variables were standardized before fitting the model to improve model convergence and ease the interpretation of model parameters.

The time of flight was not uniformly distributed between 6:30 and 12:30. Therefore it was not included in the analyses since it was arbitrarily chosen due to field conditions and integrates other sources of variability like the environmental flight conditions.

The resulting models built can be expressed as follows:

$$\ln\left(\frac{R}{1-R}\right)=a+b*Height+c*Wind\;speed+d*Cloud\;cover+e*Sun\;reflection+f*Experience+\alpha_{flight}+\beta_{photo}+\gamma_{observer}$$ (3)

R was the detection rate or the level of certainty, while α_flight_, β_photo_ and γ_observer_ were the random parameters that followed a normal distribution with a null mean and a standard deviation defined by the resulting model [44,45].

The random and fixed effects were tested using a backward procedure [44] with the Akaike Information Criterion (AIC) as a measure of the relative quality of the tested models.

Analyzes were performed with the R software (R Development Core Team, 2008) using the faraway, lme4, and lattice packages.

### Correction factors

To provide a corrected estimate of the total number of animals in the population we calculated correction factors. These were defined as the inverse of the detection rate for each photo.

Correction factor 1 was calculated to estimate the total number of hippos from one picture for one specific flight. The expected result was a factor based on image detection and on the total number of hippos estimated from the 15 photos. Correction factor 1 was first calculated for each of the 2016 experimental units, and the mean was then determined as the comprehensive correction factor. This factor was also estimated for both experienced and inexperienced groups of observers.

Correction factor 2 was calculated to give an estimate of the total population by taking into account hippos that might be out of the limited area (yellow circle, Fig 3) at the time of the survey. The added value of factor 2 comes from the inclusion of in and out movements. It was calculated using the same method as factor 1 but considered the total number of hippos detected on all pictures. Again, the correction factor was split between experienced and inexperienced observers. We then compared the two correction factors with a t-test.

Finally, we determined the impact of increasing numbers of both observers and images to get the closest estimate of the actual population while being cost effective. From the entire detection database, we randomly extracted 30 combinations of detection rates from 1 to 3 observers analyzing 1 to 10 photos. The highest rate for each combination was selected (see S1 Table). Combinations of more photos and observers would have been considered too time- and cost-consuming to implement during a survey. The process was repeated 100 times for each combination, and we then calculated the mean of the best detection rates for every association. Results were presented as a matrix of correction factor (S1 Table) and the error associated with the different combinations plotted.

### Data validation

The images from the second site were used as a validation dataset. The total number of hippos in Wilibadi II school was estimated using the same method over two flights with an experienced observer. The differences between the totals, with the two correction factors and the theoretical total numbers estimated previously, were compared with t-tests.

## Results

### Estimation of the total number of hippos

From the 8 flights over the Dungu school, the population was estimated between 55 and 61 individuals (Table 2). The estimation process took approx. one hour per flight.

**Table 2:** Estimated total number of hippos in the Dungu school for each flight.

N° flight	1	2	3	4	5	6	7	8
**Date**	1^st^ May	7 May	7 May	8 May	8 May	9 May	9 May	10 May
**Take off time**	10:10	7:55	11:30	6:50	8:00	7:20	8:45	6:45
**Total number of hippos estimated**	57	60	58	61	59	55	57	57

### Observers' counts

Most observers took approximately 8 hours to scan the 252 images, but two observers took much longer (from 10:00 to 12:45 hours) (Table 3). The three observers who took the shortest time were the ones familiar with the method and the software. The average time needed to review one photo was 129±24 secs.

**Table 3:** Detection rates and levels of certainty for each observer.

		Experienced observers			Inexperienced observers				
		1 (exp.)	2 (exp.)	3 (exp.)	4	5	6	7	8
	**Total number of animals counted**	12,734	13,199	12,515	12,509	12,199	11,371	11,505	11,864
	**Total time**	8:00	7:45	8:00	8:45	10:00	12:45	8:30	8:30
	**Average time per photo (sec)**	114	111	114	125	143	182	121	121
**DETECTION RATE**	**Mean (CI 95%)**	0.869 [0.858; 0.881]	0.902 [0.892; 0.911]	0.854 [0.843; 0.866]	0.854 [0.843; 0.865]	0.833 [0.820; 0.845]	0.776 [0.762; 0.79]	0.785 [0.773; 0.798]	0.810 [0.798; 0.821]
	**Minimum**	0.527	0.564	0.517	0.564	0.397	0.414	0.483	0.436
	**Maximum**	1.033	1.035	1.035	1.123	1.018	0.967	1.000	1.000
**LEVEL OF CERTAINTY**	**Mean (CI 95%)**	0.877 [0.869; 0.886]	0.946 [0.941; 0.951]	0.937 [0.930; 0.943]	0.770 [0.754; 0.785]	0.919 [0.912; 0.926]	0.905 [0.896; 0.913]	0.797 [0.786; 0.809]	0.689 [0.670; 0.709]
	**Minimum**	0.612	0.764	0.706	0.279	0.610	0.702	0.487	0.000
	**Maximum**	1.000	1.000	1.000	1.000	1.000	1.000	0.982	1.000

Experienced observers scored better detection rates than inexperienced ones but differences between observers within each group remain highly significant for both parameters. Values of the detection rate were less variable (CV 12.3%) than the levels of certainty (CV 14.7%) (Table 3).

These results confirm the existence of a strong observer effect during the counts: differences in DR and LC between observers are important. Some observers detected animals easily and more accurately than others even if observers with the highest LC did not always have the best DR, and the opposite was also true.

### Modeling the parameters’ effects on the counts

AIC *Likelihood Ratio Tests* showed that the three random factors (flight, photo and observer) significantly improved both the detection rate and the level of certainty. Consequently they should be used in the final models.

Flight heights were extracted from the GPS data. Once they had been sorted into classes matching the theoretical values of the flight plan, we found that flight height classes did not produce better models. Therefore, single values were then kept for further analysis and standardized in order to compare its effect with these of other factors.

We found differences in the estimated flight and observer parameters between the detection rate (DR) and level of certainty (LC) models (Table 4). Wind speed had no significant effect and was removed from both models (Table 4).

**Table 4:** Estimated parameters of the detection rate and level of certainty models.

Models	a	b	d	e	f	Std. α_flight_	Std. β_photo_	Std. γ_observer_
**Detection rate**	2.36659***	-0.07145**	-0.74768*	-0.61517***	-0.51469***	0.4414	0.3142	0.2101
**Level of certainty**	2.79614***	-0.11748***	-0.48934**	-0.27579***	-0.93356*	0.1833	0.2618	0.5618

These parameters can be included in the first equation as a + b*Height + d*Cloud cover + e*Sun reflection + f*Experience + αflight + βphoto + γobserver.

* P<0.05

**P<0.01

***P<0.001

Cloud cover and sun reflection were both sorted into 3 categories. However, in both models the middle categories had very little effect and therefore we regrouped these factors into two categories. For both models, scattered clouds were considered as no clouds without much effect upon the models. Minor sun reflection had minimal effect in the DR model, and was grouped with significant reflection for the LC model.

The models show that sun reflection has a highly significant negative effect on both the detection rate of hippos and observer confidence (Table 4). Complete cloud cover also significantly affects the LC, while its effect is less significant for the DR. Both DR and LC decrease with increasing flight height, although it is more significant for LC. Observer experience affects both models, being highly significant for DR but less important for LC.

Sun reflection was strongly correlated with acquisition time of the images (r: 0.60; p-value < 0.001).

### Determination of correction factors

Correction factors 1 and 2 show highly significant differences in all cases (Table 5, all p-values<0,001).

**Table 5:** Mean values of the correction factors and their 95% confidence intervals for the different categories of observers.

Correction factors	All observers	Experienced observers	Inexperienced observers
**1** (flight count)	1.218 [1.211; 1.226]	1.156 [1.146; 1.166]	1.256 [1.245; 1.266]
**2** (maximum count of all flights)	1.286 [1.276; 1.295]	1.220 [1.207; 1.232]	1.325 [1.312; 1.338]

**Fig 5:**
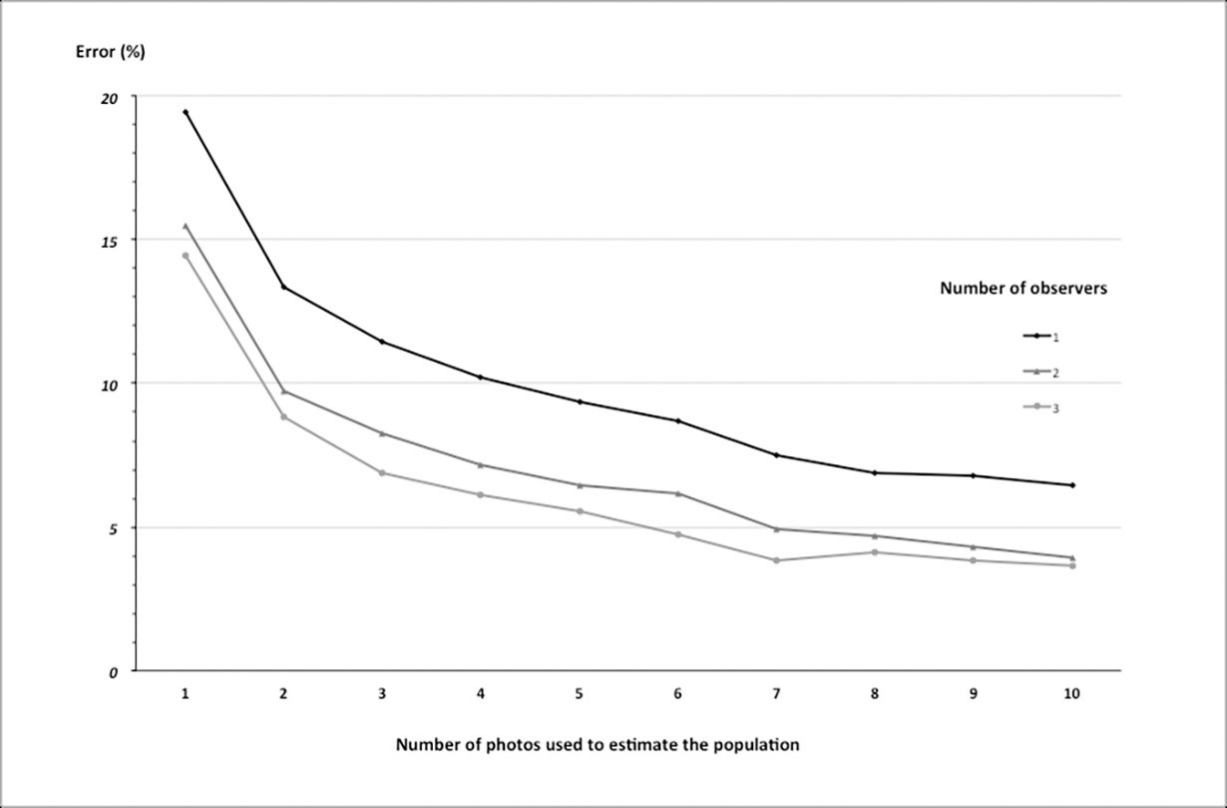
Error in the population size estimates in relation to numbers of photos and observers.

We then tried to find the ideal combination of observers and photos to give the most accurate and cost-effective population estimate. The error decreased as the number of images and observers increased. For example, with a single observer and one photograph the error was 19%, and adding another observer and another picture reduced the error by half (Fig 5). However, the improvement leveled off rapidly. Fig 5 illustrates how one can select the optimum combination to achieve minimal error (< 5%) in the population estimate. In this case, the most cost-effective combination was 2 observers reviewing 7 photos (Fig 5).

### Validation of the method

The Wilibadi II population was larger relative to the Dungu site. It was spread over a larger area and therefore at the lowest flight height two successive overlapping photos were merged to cover the entire study site. The total estimates for the two flights were 138 and 144 individuals respectively. No significant differences were found between photo counts taken at different altitudes. Almost all pictures of the second flight were partially covered by a large patch of sunlight. In consequence, estimates were low compared to the total population, with a high variance. Flight 1 was conducted under good conditions and therefore the counting was optimal. Previously, experienced observers detected 86.5% (95% CI = ±0.76%) of the hippos on average. On this new dataset, we detected 84.3% (95% CI = ±1.84%). The application of the first correction factor to the second site led to a slight underestimate of hippos by 2.5% (95% CI = ±2.1%). The use of correction factor 2 to estimate the total population with the same dataset was not significantly different and therefore validates this factor for experienced observers.

## Discussion

All detection rates calculated and analyses performed here were based on a ground truth that was itself an estimate. However, except in the case of an enclosed well-known group, it is rarely possible to know the real number of animals in the wild. The method used to estimate the total number of hippos during one flight was consistent and was easy to implement. Delvingt [7] recommended the use of multiple aerial images, and we used 15 successive pictures which covered a period longer than usual diving time. During our tests we also tried taking 20 min videos of the school to count the hippos. However, it proved harder for the observers to follow the constant movements of multiple animals at the same time on the clips, and we ended up making more mistakes. Our method showed good results, though it would be interesting to use a multicopter hovering over the hippos taking more frequent still images in order to get an even more accurate count to estimate the correction factor. We also used the hypothesis of no in-and-out movements within the group during each flight. However, differences of a few hippos were observed between flights, even during the same day. This is consistent with the group stability observed over a period of 1 to 5 days [7].

Both DR and LC were affected by increasing flight height, although weakly and inconsistently. Flight height negatively affected the confidence observers have in their detection, rather than detection itself. The effect of altitude in the validation dataset was less important, despite altitudes ranging up to 250 m. This can be explained by the high resolution of the pictures (from 0.8 cm/pixel at 40 m to 4.9 cm/pixel at 250 m), which allowed one to zoom in for more accuracy. However, we would not recommend using the maximum altitude during a large-scale survey. Indeed, it is important to be able to rapidly detect animals, even isolated individuals, in order to ensure the reviewing process is time- and cost-effective. An average height of 150m is a good compromise as it allows the detection of animals without zooming in, covers an area of over 2 hectares in one frame, and is fairly accurate when counting large groups.

Amongst the three weather factors studied, wind speed did not have much influence on the counts. However, we would recommend flying under light wind (1 to 3 m.s^-1^) as UAV performance suffers with strong wind (> 3 m.s^-1^). Sun reflection reduced the detection of hippos and increased the level of uncertainty. Indeed, broad patches of sunlight can hide large parts of the river and make hippos very hard—if not impossible—to see. It is interesting to note that for DR the second class of sun reflection could be considered as having the same effect as no sun at all, meaning only big patches matter, while for LC smaller patches affected the confidence more. The negative effect of sun reflection reinforces the call to fly at lower wind speeds. Indeed, high wind speeds can lead to disrupted water surfaces, and more reflection at the water surface. Moreover, the camera’s orientation at the time of image capture can make surface reflection worse in images. The use of a gimbaled payload could help with this last point by ensuring all photos are nadir. Cloud cover showed a slight effect on detection. For both DR and LC, the middle class of cloud cover (scattered clouds or light cover) was classified as no clouds as it was not significant in the models. The negative effect of both cloud cover and sun reflection may seem to counter-balance each other. However, heavy cloud cover can have the same effect as the sun by creating dark spots on the pictures where animals are hard to detect. Therefore, we recommend flying when sun is still low on the horizon and when there is no cloud cover, or else when cloud cover is light and even. Since large patches of sun reflection depend on the time of day, this last point emphasizes the recommendation for early flights [7]. Indeed, no sun reflection was noticed before 8:45. Flights should then be conducted between 6:30 and 8:45 in our study area (3–4° N; 29–30° E).

The counts showed a significant difference between inexperienced and experienced observers. The models show that experience played a prominent role in the DR. On the other hand, it was less important for the LC. Experienced observers achieved better detection rates and were generally more confident in their detection while the LC of the novices was more variable. Experienced observers therefore need a smaller correction factor to estimate the total population. Moreover, an observer’s experience does not need to be extensive to make a difference, as observers 1 and 3 were only trained for a short time. It was demonstrated previously during traditional surveys that a little field experience is needed [18].

When applied to data from the second site reviewed by the most trained observer, correction factors 1 and 2 approximated well the number of hippos. The value of correction factor 2 (1.22) is very close to the value found by Delvingt [7] in Virunga (1.25). These results confirm the use of correction factor 2 for hippo surveys, regardless of the study site, as it accounts for the behavior of this species.

Fig 5 shows that adding one picture and one observer dramatically improved the quality of the counts. Furthermore, increasing the number of photos reduced the counting errors more than increasing the number of observers. Therefore, to reduce observer bias, we suggest selecting and training just a couple of observers, rather than investing in more observers. These trained observers could then calculate their own correction factors with a dataset from a specific site. By then applying their factors to pictures of a total survey (one or two passes of the UAV with picture overlap) one would reduce the risk of errors. The huge amount of data and the need for multiple observers to minimize the observer effect are often described as an important drawback of UAS in wildlife monitoring [27]. Our results highlight the option of reducing time and costs by investing a little time (for example, two weeks) in the training of two observers who can then apply their expertise at different sites.

## Conclusion and perspectives

This work focused on optimizing the visibility and detection of hippos from high-resolution images. It also evaluated the proportion of hippos detected in order to generate a correction factor that will improve the accuracy of future population estimates. The results are conclusive and the method can now be applied on a larger scale. UAV flight plans are easy, accurate and reproducible and can be used to collect a series of standardized hippo estimates that are comparable. UAS are perfectly suitable for surveying rivers and pools at low cost. The application of the first correction factor would give a good approximation of the actual hippo population but the application of correction factor 2 could give a more accurate estimate by taking into account those roaming outside the main school or hiding under riverine trees. The application of correction factor 1 or 2 can be left to the decision of the managers depending on how conservative the estimate is required to be.

This study casts new light on a method for monitoring a species that is usually neglected when it comes to wildlife counts. Drone technology is expected to evolve rapidly in the years to come, making UAS more reliable, efficient, and cheaper [46]. Therefore UAS will become a very useful and affordable survey tool for other species requiring specific monitoring.

## Supporting information

S1 Table Simulation of randomly selected observers and pictures. (XLSX)

S2 Table Complete dataset. Flight parameters and counts from the different observers are associated to each photo.
(XLSX)
